# Refractive surprise of irregular astigmatism following cataract surgery in two patients with neglected subclinical corneal ectasia: two case reports

**DOI:** 10.1186/s12886-023-02984-4

**Published:** 2023-05-29

**Authors:** Leran Chen, Xiaoyong Chen, Fanshu Li, Ziyuan Liu

**Affiliations:** 1 Department of Ophthalmology, Peking University Third Hospital, Beijing key laboratory of restoration of damaged ocular nerve, Beijing, 100191 China; 2 Peking University Institute of Laser Medicine, Beijing, China

**Keywords:** Subclinical corneal ectasia, Astigmatism, Cataract surgery, Steep-axis incisions

## Abstract

**Background:**

Corneal ectatic diseases are a group of corneal disorder characterized by the steepening and thinning of the cornea. Older people are not a high-risk population for corneal ectatic diseases; due to the lack of typical preoperative topographic manifestations, there is a high possibility that corneal ectasia is undetected.

**Case presentation:**

Two patients with subclinical corneal ectasia and senile cataracts presented with irregular astigmatism after steep-axis incision during cataract surgery. The two cases presented in this case report are rare because both patients experienced tremendous changes in astigmatism after cataract surgery.

**Conclusion:**

This case report may shed some light on astigmatism-correcting steep-axis incisions in cataract surgeries.

## Background

Corneal ectasia is a group of progressive diseases characterized by corneal steepening and thinning. Degradation can occur automatically or can be surgically induced. Corneal ectasia usually appears in adolescent patients and gradually progresses until approximately 40 years of age. However, it is possible for corneal ectasia to progress slightly after the age of 40 years—the progression is slower in steeper corneas [1]. Subclinical corneal ectasia lacks topometric or tomographic manifestations, and densitometry and biomechanical indices should be combined for early detection [2]. This case report outlines two patients with subclinical corneal ectasia with irregular astigmatism that increased after cataract surgery; it offers experience for correcting astigmatism in cataract surgeries.

## Case Presentation

### Case 1

**Fig. 1:**
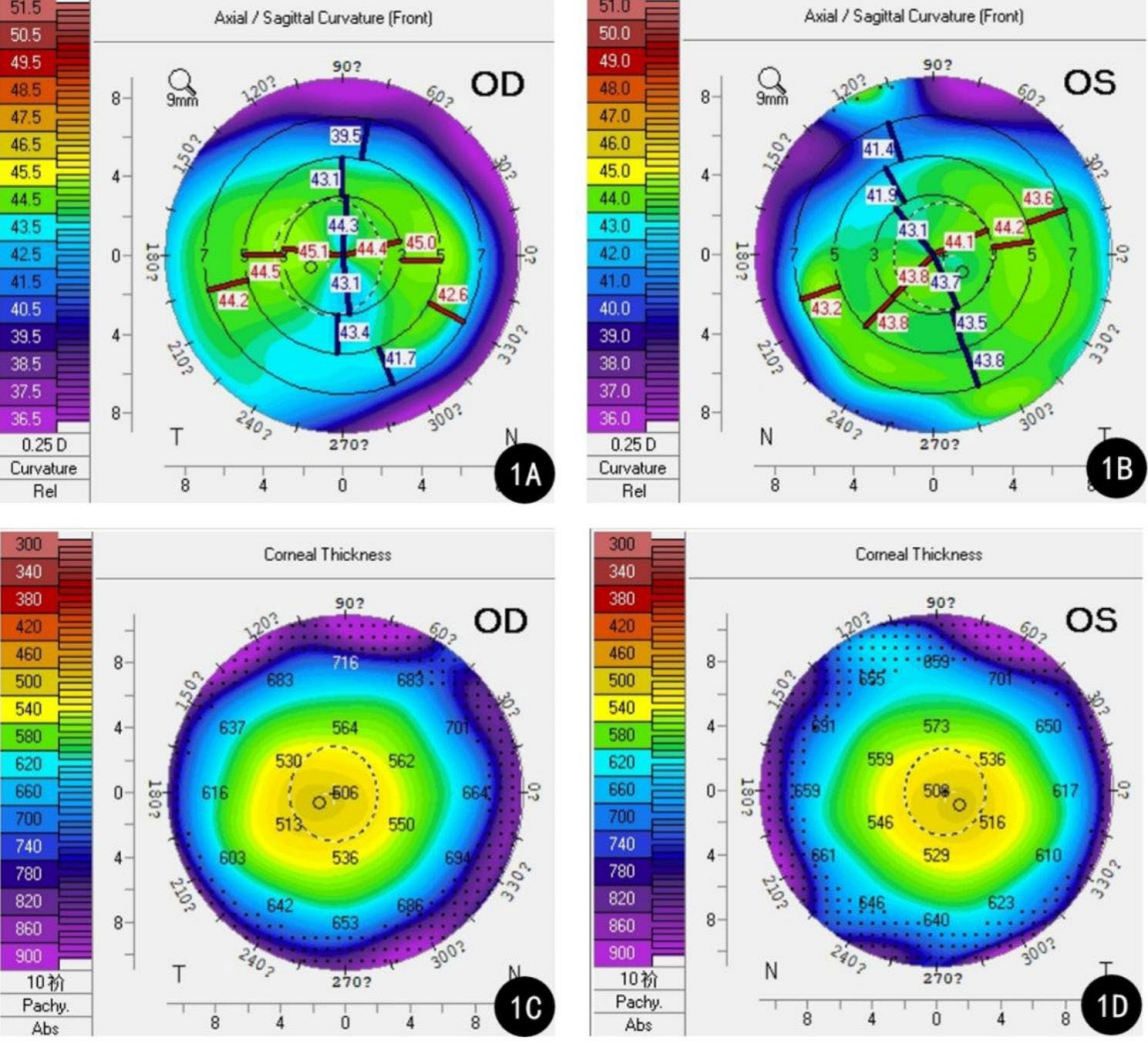
Right and left Pentacam topographic patterns from Case 1. **A**: The sagittal topographic map in the right eye; **B**: The sagittal topographic map in the left eye; **C**: The corneal thickness map in the right eye; **D**: The corneal thickness map in the left eye

**Fig. 2:**
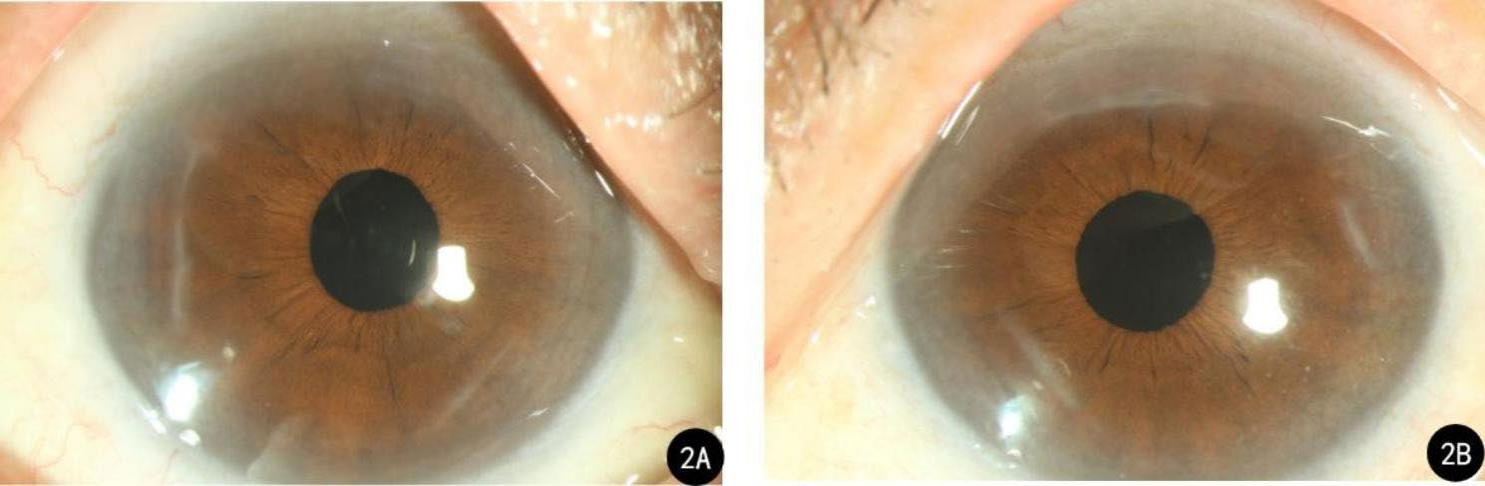
Right and left postoperative anterior segment photography from Case 1. **A**: Postoperative anterior segment photography of the right eye; **B**: Postoperative anterior segment photography of the left eye

A 73-year-old female was admitted to our hospital for cataract surgery. On admission, both lenses were clouded, with a visual acuity of 20/60 in the right eye and 20/130 in the left eye. Preoperative keratometry and biometry data were collected using IOLMaster (IOLMaster700, Carl Zeiss Meditec AG, Jena, Germany) and Pentacam (Oculus, Wetzlar, Germany). Readings of the right eye from the IOLMaster were K1 = 43.37 diopters (D), K2 = 45.00D, axial length (AL) = 23.90 mm, and anterior chamber depth (ACD) = 2.50 mm. The corresponding figures in her left eye were K1 = 43.25D, K2 = 44.12D, AL = 23.84 mm, ACD = 2.34 mm. Pentacam showed a skewed radial axis and paracentral steepening on the sagittal topographic map in both eyes (Fig. 1A-B). The simulated keratometry (Sim K) readings presented by Pentacam were K1 = 43.6D, K2 = 44.8D in the right eye and K1 = 43.2D, K2 = 43.9D in the left eye. Sim K astigmatism (Astig.) was 1.2D, total corneal irregular astigmatism (WFA HO RMS) (4 mm zone) was 0.211 μm, the posterior–anterior corneal radius ratio (B/F ratio) was 82.2%, and central corneal thickness (CCT) was 506 μm. The parameters of the left eye were Sim K K1 = 43.2D, K2 = 43.9D, Astig.=0.5D, WFA HO RMS = 0.109 μm, B/F ratio = 82.4%, and CCT = 508 μm (Fig. 1C-D). Since the incidence of dry eye in patients with senile cataract are higher than the general population, and the presence of dry eye disease may lead to inaccuracy in measured astigmatism, the patient was screened for dry eye disease [3, 4]. The patient had negative corneal fluorescein staining and intact corneal epithelia. As the patient presented with against-the-rule astigmatism (i.e., horizontal meridian steeper), an uneventful cataract surgery was performed with two symmetric incisions on the steep axis to correct the astigmatism (Fig. 2A-B).

**Fig. 3:**
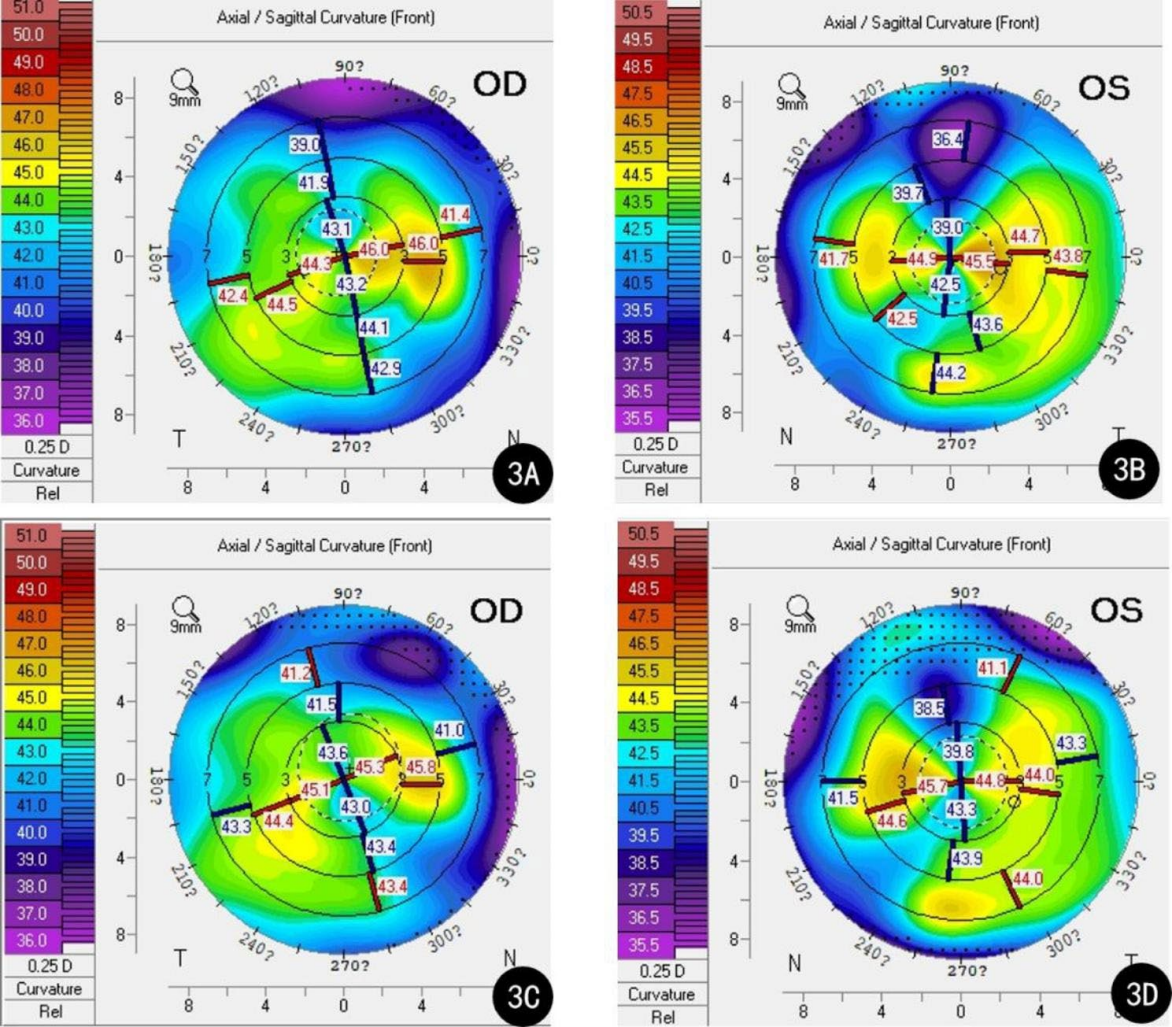
Right and left postoperative Pentacam sagittal curvature maps from Case 1. **A**: The right sagittal curvature map one month after the surgery; **B**: The left sagittal curvature map one month after the surgery; **C**: The right sagittal curvature map 3 months after the surgery; **D**: The left sagittal curvature map 3 months after the surgery

The patient’s visual acuity was not significantly improved in either eye after surgery; it was 20/40 in the right eye and 20/170 in the left eye on the first postoperative day. Slit lamp examination showed no signs of corneal edema or a dislocated intraocular lens, and no significant abnormalities were observed on fundus examination. One month and 3 months after surgery, her visual acuity remained unimproved. Postoperative examination at one month post-surgery using Pentacam showed an increase in astigmatism: K1 = 43.0D, K2 = 45.3D, Astig.=2.3D in the right eye; K1 = 40.8D, K2 = 45.4D, Astig.=4.6D in the left eye (Fig. 3A-B). At the 3-month follow-up, her parameters on Pentacam were K1 = 43.1D, K2 = 45.1D, Astig.=2.0D in the right eye; K1 = 41.6D, K2 = 45.2D, Astig.=3.6D in the left eye (Fig. 3C-D).

**Fig. 4:**
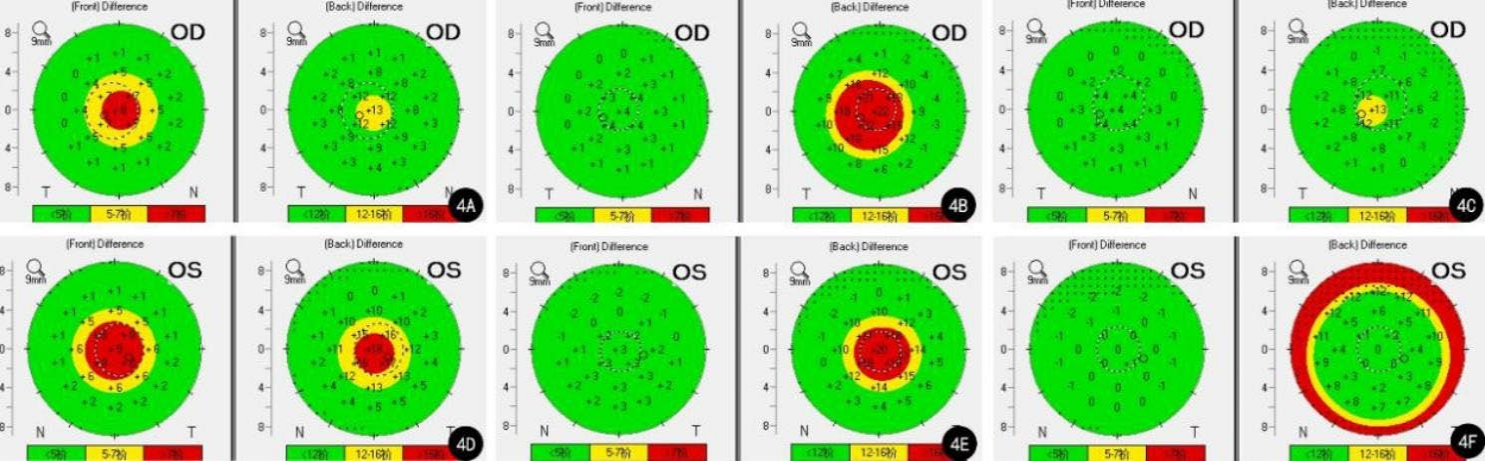
Right and left elevation maps on the BAD from Case 1. **A**: Preoperative elevation map in the right eye; **B**: The right elevation map one month after the surgery; **C**: The right elevation map 3 months after the surgery; **D**: Preoperative elevation map in the left eye; **E**: The left elevation map one month after the surgery; **F**: The left elevation map 3 months after the surgery

A review of the patient’s preoperative topography data showed significant abnormalities on the Belin–Ambrósio enhanced ectasia display (BAD), demonstrating an elevation from the best-fit sphere (BFS) in both the anterior and posterior corneal surfaces. Df (deviation of normality of the front elevation) was 3.43, Db (deviation of normality of the back elevation) was 1.78, and the final D index was 2.40 in the right eye; in the left eye, the Df = 4.17, Db = 2.99, and D = 2.69 (Fig. 4A, D). Moreover, topometric indices showed symmetrical aberrations in the patient’s cornea. The index of surface variance (ISV) was 41, index of vertical asymmetry (IVA) was 0.24, keratoconus index (KI) was 1.10, and topographical keratoconus classification (TKC) was KC1 in the right eye. The indices in the left eye were ISV = 38, IVA = 0.34, KI = 1.10, and TKC = KC1.

At 1 month postoperatively, the BAD parameters were as follows: Df = 0.85, Db = 4.23, D = 4.16 in the right eye; Df = 0.54, Db = 3.56, D = 4.80 in the left eye (Fig. 4B, E). The corresponding figures at 3 months postoperatively were Df = 1.20, Db = 1.74, D = 2.44 in the right eye; Df=-1.46, Db = 9.13, D = 3.69 in the left eye (Fig. 4C, F). The BAD indices of the patient showed that both her anterior corneal surfaces returned to normal, while her posterior corneal surfaces were elevated compared to the BFS 3 months after the surgery. The indices on the topometric display at the 1 month follow-up were as follows: ISV = 42, IVA = 0.34, KI = 1.12, TKC = KC1 in the right eye; ISV = 56, IVA = 0.52, KI = 1.12, TKC = KC1-2 in the left eye. Three months postoperatively, the indices were as follows: ISV = 42, IVA = 0.37, KI = 1.09, TKC = KC1 in the right eye; ISV = 40, IVA = 0.36, KI = 1.05, and TKC was normal in the left eye. The patient did not undergo further follow-up examinations.

### Case 2

**Fig. 5:**
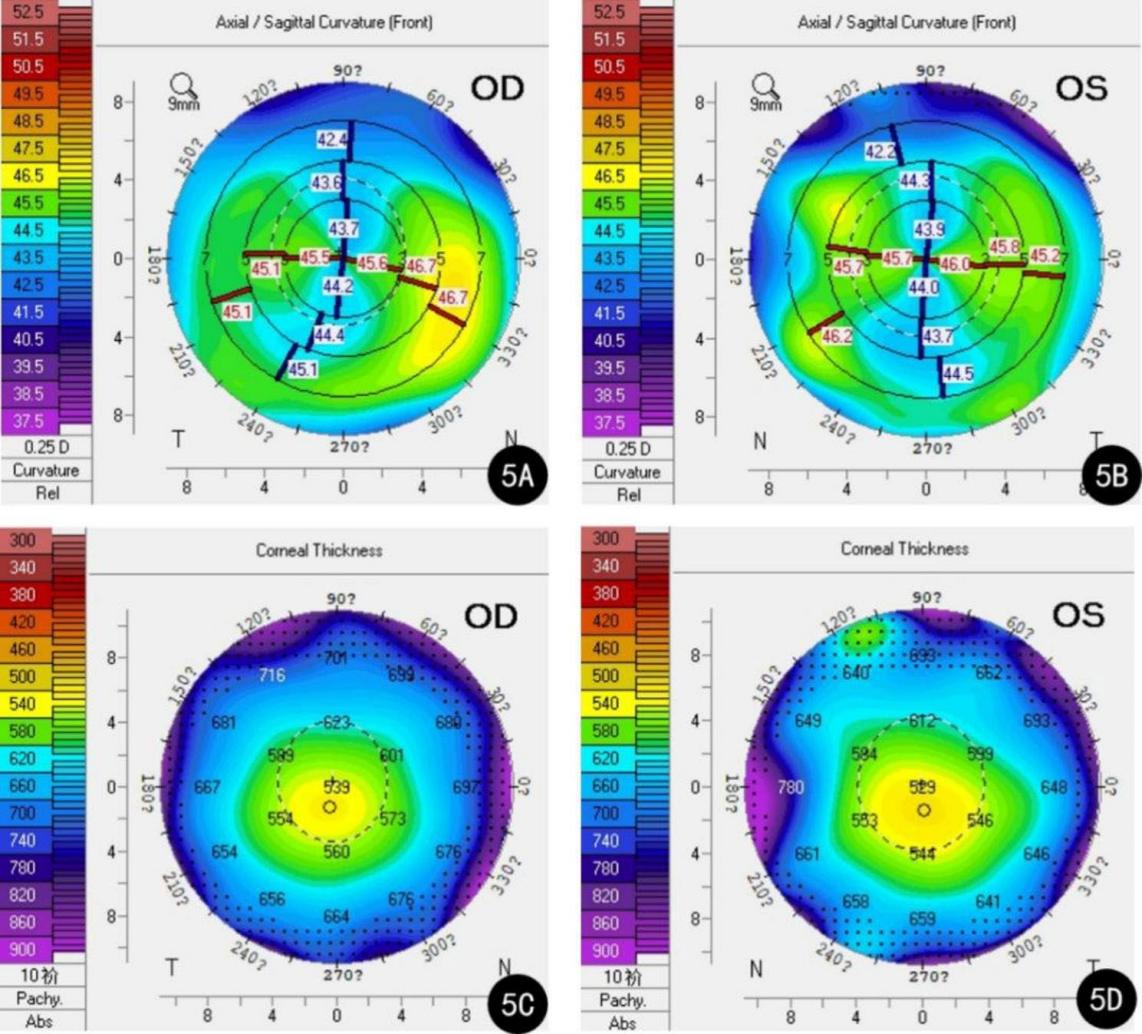
Right and left Pentacam topographic patterns from Case 2. **A**: The sagittal topographic map in the right eye; **B**: The sagittal topographic map in the left eye; **C**: The corneal thickness map in the right eye; **D**: The corneal thickness map in the left eye

**Fig. 6:**
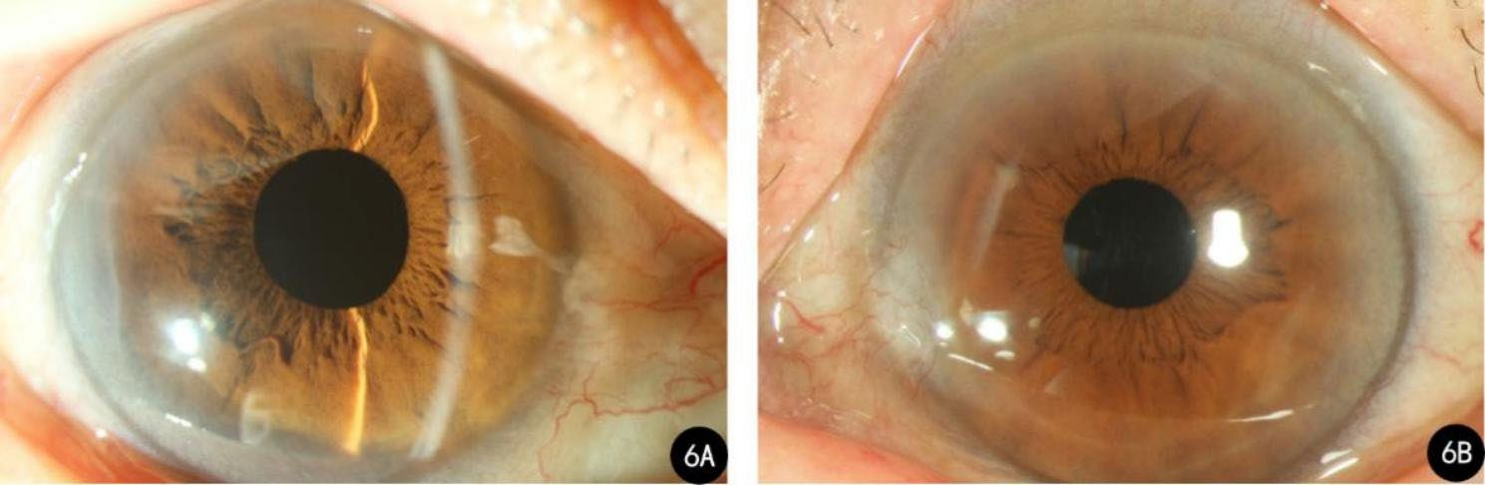
Right and left postoperative anterior segment photography from Case 2. **A**: Postoperative anterior segment photography of the right eye; **B**: Postoperative anterior segment photography of the left eye

A 73-year-old female presented to our clinic with a history of cataract surgery. Preoperative examination revealed clouded lenses in both eyes, and her visual acuity was 20/70 in the right eye and 20/50 in the left eye. The patient’s keratometry and biometry readings on the IOLMaster were: right eye—K1 = 43.95D, K2 = 45.57D, AL = 23.47 mm, ACD = 3.12 mm; left eye—K1 = 43.77D, K2 = 46.18D, AL = 23.25 mm, ACD = 3.05 mm. A topographic examination was performed using Pentacam, and the sagittal topographic maps of her right and left eye also revealed a crab claw pattern (Fig. 5A-B). Her keratometry data on Pentacam were as follows: right eye—K1 = 43.9D, K2 = 45.5D, Astig.=1.9D, WFA HO RMS = 0.248 μm, B/F ratio = 79.8%, CCT = 539 μm; left eye: K1 = 43.9D, K2 = 45.8D, Astig.=1.9D, WFA HO RMS = 0.248 μm, B/F ratio = 80.0%, CCT = 529 μm (Fig. 5C-D). Her corneal fluorescein staining results were negative and her corneal epithelia were intact. Two symmetric incisions on the steep axis in phacoemulsification were performed smoothly (Fig. 6A-B).

On postoperative day 1, visual acuity was 20/25 in the right eye and 20/50 in the left eye. One month later, her best-corrected visual acuity (BCVA) in the right eye was 20/25 with − 0.75 -2.00 × 130, and 20/40 with + 1.25 -4.00 × 110 in the left eye. Anterior segment photography showed that Grade 1 nasal pterygium was present in both eyes of this patient (Fig. 6). Pterygium was found to be positively correlated with corneal astigmatism, but pterygium does not tend to induce astigmatism until it reaches a certain size [5, 6]. Avisar et al. [7] reported that lesions exceeding 16% of the corneal radius or 1.0 mm in size induce significant astigmatism. Since the nasal pterygium in the patient was small in size, extending approximately 14% and 4% of the corneal radius in the right and left eye, the influence of nasal pterygium on corneal astigmatism in this case was considered limited.

**Fig. 7:**
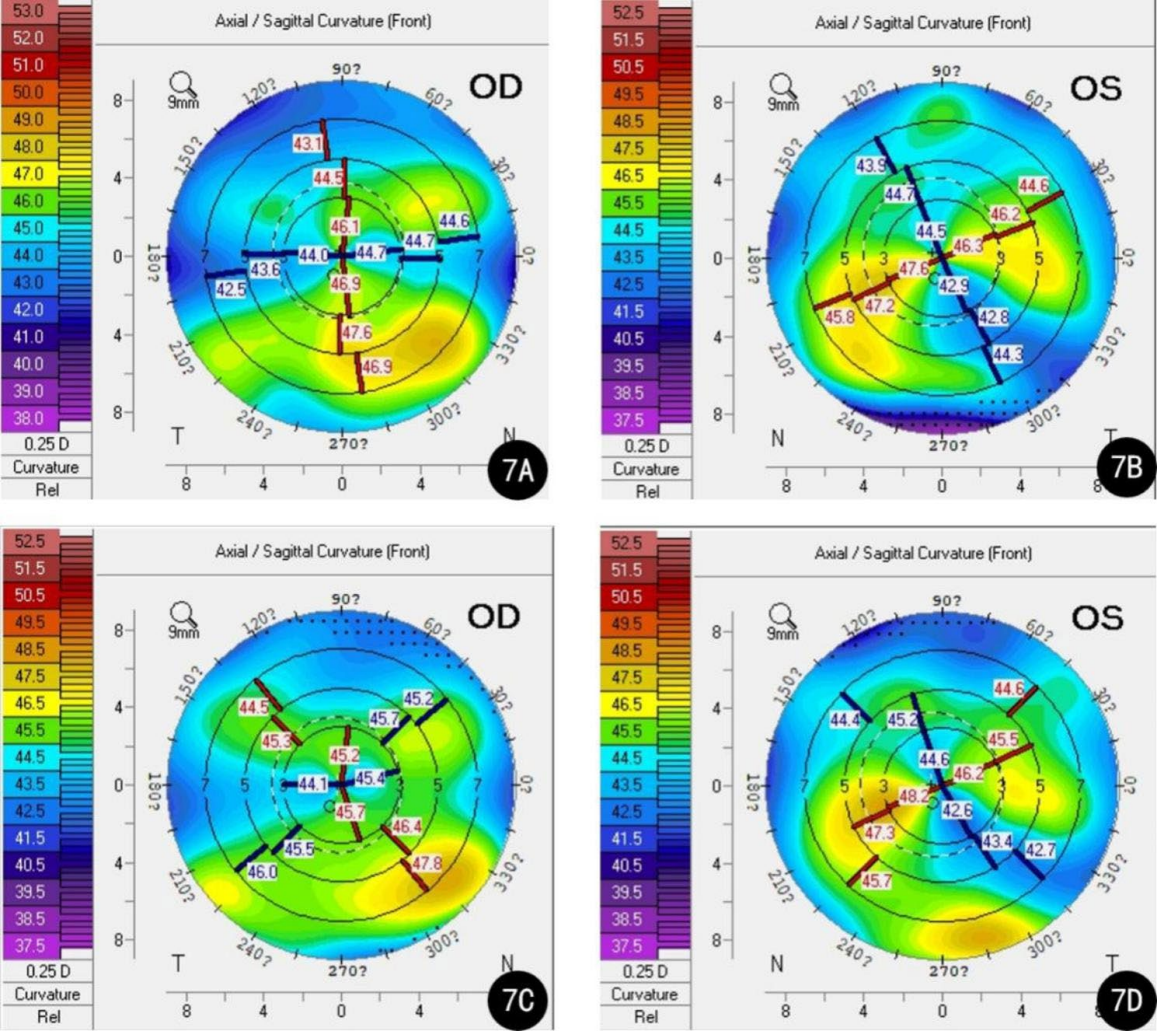
Right and left postoperative Pentacam sagittal curvature maps from Case 2. **A**: The right sagittal curvature map one month after the surgery; **B**: The left sagittal curvature map one month after the surgery; **C**: The right sagittal curvature map 3 months after the surgery; **D**: The left sagittal curvature map 3 months after the surgery

Her keratometry data on Pentacam 1 month after the surgery were as follows: right eye—K1 = 44.2D, K2 = 46.4D, Astig.=2.2D; left eye—K1 = 43.8D, K2 = 47.0D, Astig.=3.2D (Fig. 7A-B). Three months after the surgery, her BCVA was 20/25 with − 0.25–0.25 × 80 in the right eye, and 20/28 with + 1.00 -3.50 × 108 in the left eye. Keratometry: right eye—K1 = 44.4D, K2 = 45.4D, Astig.=1.0D; left eye: K1 = 43.6D, K2 = 47.0D, Astig.=3.3D (Fig. 7C-D). No corneal edema or dislocated IOL was observed on follow-up slit-lamp examinations, and no abnormality was found on fundus examination.

**Fig. 8:**
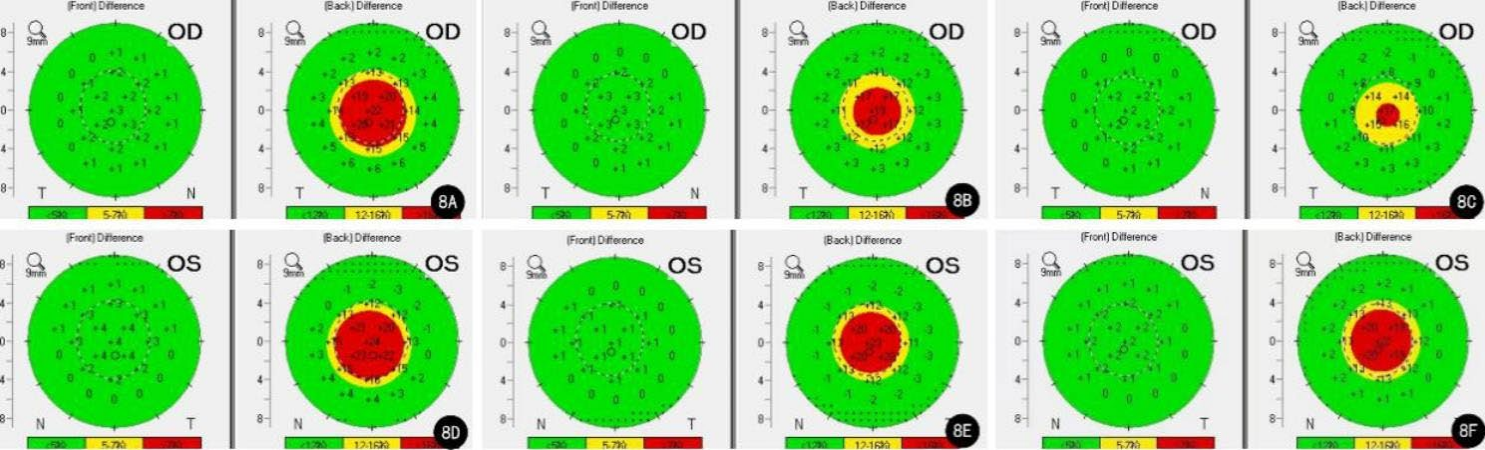
Right and left elevation maps on the BAD from Case 2. **A**: Preoperative elevation map in the right eye; **B**: The right elevation map one month after the surgery; **C**: The right elevation map 3 months after the surgery; **D**: Preoperative elevation map in the left eye; **E**: The left elevation map one month after the surgery; **F**: The left elevation map 3 months after the surgery

Abnormalities in BAD and topometric indices were neglected in the preoperative Pentacam examination. Her BAD indices were: right eye—Df=-0.08, Db = 4.05, D = 2.73; left eye—Df = 0.99, Db = 4.59, D = 3.01 (Fig. 8A, D). The abnormal topometric parameters were as follows: right eye— ISV = 25; IVA = 0.23; KI = 1.06; left eye— ISV = 25, IVA = 0.17, KI = 1.06; TKC was normal in both eyes.

After surgery, the BAD revealed that irregularities in the patient’s posterior corneal surfaces remained. At 1 month postoperatively, the BAD parameters were as follows: right eye—Df = 0.36, Db = 3.21, D = 2.43; left eye—Df=-0.94, Db = 4.24, D = 3.21 (Fig. 8B, E). The BAD indices at 3 months postoperatively were as follows: right eye: Df=-0.37, Db = 2.70, D = 2.35; left eye: Df=-0.22, Db = 3.99, D = 3.35 (Fig. 8C, F). The indices on the topometric display showed slight fluctuations in corneal symmetry. One month after surgery, the topometric indices were as follows: right eye—ISV = 32; IVA = 0.32; KI = 1.09; TKC = KC1; left eye—ISV = 28, IVA = 0.20, KI = 1.00, TKC was normal. Three months later, the corresponding parameters were as follows: right eye—ISV = 25, IVA = 0.24, KI = 1.07, and TKC was normal; left eye—ISV = 29, IVA = 0.25, KI = 1.05, and TKC was normal. The patient wore glasses to correct her visual acuity postoperatively.

## Discussion and conclusions

The two cases were similar in preoperative topography, ectasia indices, and symmetry indices, which were neglected before surgery.

First, both patients presented with a skewed radial axis and interior steepening preoperatively, resulting in the appearance of a crab claw pattern on the sagittal topographic map. Trattler [8] discovered that in patients with astigmatism and a crab claw pattern, limbal relaxing incisions (LRIs) or astigmatic keratotomy (AK) on the steep axis may lead to an increase in irregular astigmatism postoperatively. The crab claw pattern, also known as butterfly or pellucid pattern, demonstrates flattening of the cornea along the vertical axis and steepening of the inferior corneal periphery [9]. The crab claw pattern is a classic pattern of pellucid marginal corneal degeneration (PMD), but can also be seen in patients with other types of corneal ectasia. Lee et al. [10] and Koc et al. [11] found that patients with crab claw patterns were more likely to develop keratoconus than those with PMD. Research has also shown that crab claw patterns also appear on the sagittal power maps of patients with Mooren ulcer and that patients with a crab claw pattern have greater astigmatism than those with other patterns [12]. Thus, a crab claw pattern on topography suggests that the patient might have a certain ectatic corneal disease [13]. For patients with a crab claw pattern, more examinations should be performed for differential diagnosis, such as a full 12 mm corneal thickness map, Scheimpflug images, and tomographic and densitometric evaluations [11].

There are currently no diagnostic metrics for corneal ectasia, but guidelines from the American Academy of Ophthalmology (AAO) suggest that there is usually a steepening in the central or central peripheral cornea on patients’ topography [14]. Patients with corneal ectasia share common topographical features. An asymmetric island pattern is often seen on elevation maps, and thickness maps reveal that the thinnest point of the ectatic cornea is usually located peripherally [15, 16]. With these features present in both cases, it is possible that the two patients reported above had subclinical ectatic corneal diseases.

In patients with subclinical degradation or susceptibility to biomechanical changes, there is a possibility of postoperative corneal ectasia and astigmatism increase, even without significant irregularities on the corneal surface. For patients with higher levels of ectasia susceptibility, any biomechanical disturbance, such as eye rubbing or incisions, might lead to serious corneal irregularities [17]. However, subclinical degradation and ectasia susceptibility are easily overlooked, and patients with high susceptibility to corneal ectasia may not be detected until after cataract surgery. Preoperative geometric data should be thoroughly examined and integrated with intraoperative parameters to screen for subclinical corneal ectasia [18]. Therefore, for patients without abnormalities on the pre-cataract display, more attention should be paid to other displays such as BAD and topometric display [19]. In addition to the crab claw pattern on sagittal power maps, both cases also presented aberrations in the BAD and topometric indices. The BAD combines enhanced elevation data and pachymetry data, and detects mild corneal ectasia by comparing the patient’s cornea with a standard reference shape [20]. The indices of BAD demonstrate the deviation of the cornea from the reference value. For instance, Df and Db show the deviations in the front and back elevations, respectively, and the final D is the total deviation calculated by regression analysis, suggesting the probability of corneal ectatic diseases [20]. Muftuoglu et al. [21] reported that the D index has an outstanding value in forecasting keratoconus and can be used for the differential diagnosis of clinical and subclinical keratoconus, with 100% sensitivity and specificity. However, the D index has a limited value in discriminating subclinical keratoconus from normal corneas, with a sensitivity of only 60%. It has been reported that in patients with corneal ectasia after LASIK, despite low preoperative risk, BAD can detect irregularities [22]. In general, as parameters demonstrating corneal stability and suggesting a patient’s susceptibility to corneal ectasia, BAD indices should be considered before corneal incision surgery.

The topometric display of Pentacam demonstrates symmetry of the anterior corneal surfaces [18]. Among all topometric indices, ISV reveals irregularity of the corneal surface, and this index may increase in patients with astigmatism, corneal scarring, or other factors causing corneal irregularity. IVA shows symmetry of corneal curvature, and an abnormal axis of astigmatism and corneal ectasia could both lead to an increase in IVA. KI is the ratio of the radius values in the superior and inferior corneal segments, and it increases significantly in patients with clinical keratoconus or PMD [23]. Lastly, TKC is a rating based on the topometric indices used in the diagnosis of keratoconus. Both BAD and the topometric display are able to detect mild irregularities of the cornea and might suggest the possibility of postoperative astigmatism or corneal ectasia after cataract surgery [24].

Both patients had against-the-rule astigmatism, and their incisions were placed horizontally. Postoperative corneal irregularities in patients indicate that the horizontal axis of the cornea may have a greater role in the maintenance of overall biomechanical stability, and the incision in cataract surgery is likely to interfere with stability. Wallace et al. [25] discovered that cataract surgery might lead to a reduction in corneal stiffness and faster intrinsic deformation. Although no association was found between the size of the incision and the degree of stiffness change, Chang et al. [26] found that a smaller incision helps improve astigmatism control after cataract surgery. Furthermore, the location of the incision also influences surgically induced astigmatism (SIA) in cataract surgery: temporal incisions result in the lowest SIA in patients with mild preoperative astigmatism, while superior incisions have better outcomes in patients with higher levels of preoperative astigmatism [27]. Between the two types of horizontal incisions, nasal incisions lead to a much higher SIA than temporal incisions [28]. Even with the same incision, the choice of suture in cataract surgery has an impact on the SIA level. Eslami et al. [29] reported that using X-pattern sutures in scleral tunnel incisions provides better control of the SIA in patients with mild corneal steepening. These findings suggest that the horizontal axis of the cornea might play a significant role in maintaining corneal shape and that horizontal incisions and sutures are likely to cause higher levels of astigmatism after cataract surgery.

Due to the limitations of astigmatism-correcting steep-axis incisions, other options for correction of corneal astigmatism should be taken into account. There are multiple procedures to correct astigmatism in patients with cataract, including toric intraocular lenses (IOLs) [30], keratotomy [31, 32], limbal relaxing incisions (LRI) [33], excimer laser in situ keratomileusis [34], etc. Like relaxing incisions from the steep axis, toric IOLs have been widely applied in the correction of astigmatism, especially moderate to severe astigmatism, in cataract surgeries [35]. Toric IOL implantation is popular for its effectiveness, predictability and better astigmatism control [36]. However, compared to relaxing incisions, complications like macular edema or retinal detachment may arise after toric IOL implantation [30]. Toric IOLs are likely to rotate postoperatively, and large rotations may affect the IOL power, where reposition of the IOL may be needed [37, 38]. In addition, since toric IOL implantation is an expensive procedure compared to relaxing incisions, affordability should also be taken into consideration when deciding the optimal treatment plan for patients.

In conclusion, the two patients presented in this case report indicate that subclinical corneal ectatic diseases might occur in senile patients. The corneas of these patients are biomechanically unstable, and using a steep-axis incision to correct astigmatism might interfere with mechanical stability, causing corneal deformation and extreme curvature change, leading to high levels of postoperative astigmatism. Therefore, in cases with plans to perform a steep-axis incision in cataract surgery, more attention should be paid to symmetry indices and ectatic disease screening indices in topography to wisely choose applicable cases and incision locations.

## Data Availability

All data generated or analyzed during this study are included in this published article.

## References

[1] Hashemi H, Asgari S, Mehravaran S, Emamian MH, Fotouhi A (2020). Keratoconus after 40 years of age: a longitudinal comparative population-based study. Int Ophthalmol.

[2] Augustin VA, Son HS, Baur I, Zhao L, Auffarth GU, Khoramnia R. Detecting subclinical keratoconus by biomechanical analysis in tomographically regular keratoconus fellow eyes. Eur J Ophthalmol. 2021:11206721211063740. 10.1177/11206721211063740 34841930

[3] de Paiva CS (2017). Effects of Aging in Dry Eye. Int Ophthalmol Clin.

[4] Epitropoulos AT, Matossian C, Berdy GJ, Malhotra RP, Potvin R (2015). Effect of tear osmolarity on repeatability of keratometry for cataract surgery planning. J Cataract Refract Surg.

[5] Hansen A, Norn M (1980). Astigmatism and surface phenomena in pterygium. Acta Ophthalmol.

[6] Tomidokoro A, Miyata K, Sakaguchi Y, Samejima T, Tokunaga T, Oshika T (2000). Effects of pterygium on corneal spherical power and astigmatism. Ophthalmology.

[7] Avisar R, Loya N, Yassur Y, Weinberger D (2000). Pterygium-induced corneal astigmatism. Isr Med Association journal: IMAJ.

[8] Trattler W, editor. Editor Ectasia, irregular astigmatism in patients scheduled for cataract surgery. Prepping the Ocular Surface for Refractive Cataract Surgery; 2020 January. p. 29.

[9] Maguire LJ, Klyce SD, McDonald MB, Kaufman HE (1987). Corneal topography of pellucid marginal degeneration. Ophthalmology.

[10] Lee BW, Jurkunas UV, Harissi-Dagher M, Poothullil AM, Tobaigy FM, Azar DT (2007). Ectatic disorders associated with a claw-shaped pattern on corneal topography. Am J Ophthalmol.

[11] Koc M, Tekin K, Inanc M, Kosekahya P, Yilmazbas P. Crab claw pattern on corneal topography: pellucid marginal degeneration or inferior keratoconus? Eye (London, England). 2018;32(1):11 – 8. 10.1038/eye.2017.198 PMC5770716 28937143

[12] Yoshihara M, Maeda N, Soma T, Fuchihata M, Hayashi A, Koh S (2015). Corneal topographic analysis of patients with Mooren ulcer using 3-dimensional anterior segment optical coherence tomography. Cornea.

[13] Koçluk Y, Yalniz-Akkaya Z, Burcu A, Örnek F (2015). Comparison of Scheimpflug imaging analysis of pellucid marginal corneal degeneration and keratoconus. Ophthalmic Res.

[14] Garcia-Ferrer FJ, Akpek EK, Amescua G, Farid M, Lin A, Rhee MK (2019). Corneal Ectasia Preferred Practice Pattern® Ophthalmology.

[15] Fuchihata M, Maeda N, Toda R, Koh S, Fujikado T, Nishida K (2014). Characteristics of corneal topographic and pachymetric patterns in patients with pellucid marginal corneal degeneration. Jpn J Ophthalmol.

[16] Koc M, Tekin K, Tekin MI, Uzel MM, Kosekahya P, Ozulken K (2018). An Early Finding of Keratoconus: increase in corneal densitometry. Cornea.

[17] Giri P, Azar DT (2017). Risk profiles of ectasia after keratorefractive surgery. Curr Opin Ophthalmol.

[18] Lopes BT, Ramos IC, Dawson DG, Belin MW, Ambrósio R (2016). Jr. Detection of ectatic corneal diseases based on pentacam. Z Med Phys.

[19] Donoso R, Rodríguez Á, Esteffan K, Lagos C, Aránguiz D, Hernández N (2021). Analysis of OPD-Scan and Pentacam parameters for early Keratoconus detection. Am J Ophthalmol.

[20] Belin MW, Villavicencio OF, Ambrósio RR (2014). Jr. Tomographic parameters for the detection of keratoconus: suggestions for screening and treatment parameters. Eye Contact Lens.

[21] Muftuoglu O, Ayar O, Hurmeric V, Orucoglu F, Kılıc I (2015). Comparison of multimetric D index with keratometric, pachymetric, and posterior elevation parameters in diagnosing subclinical keratoconus in fellow eyes of asymmetric keratoconus patients. J Cataract Refract Surg.

[22] Ambrósio RJr, Dawson DG, Salomão M, Guerra FP, Caiado AL, Belin MW (2010). Corneal ectasia after LASIK despite low preoperative risk: tomographic and biomechanical findings in the unoperated, stable, fellow eye. J refractive Surg (Thorofare NJ: 1995).

[23] Shen J, Li H, Chen Y, Liu L, Cui H (2023). Clinical observations of corneal topographic and tomographic changes in congenital ptosis eyes: a study in China. Int Ophthalmol.

[24] Hashemi H, Beiranvand A, Yekta A, Maleki A, Yazdani N, Khabazkhoob M (2016). Pentacam top indices for diagnosing subclinical and definite keratoconus. J Curr Ophthalmol.

[25] Wallace HB, Misra SL, Li SS, McKelvie J (2019). Biomechanical changes in the cornea following cataract surgery: a prospective assessment with the corneal visualisation Scheimpflug Technology. Clin Exp Ophthalmol.

[26] Chang SW, Su TY, Chen YL (2015). Influence of ocular features and incision width on surgically induced astigmatism after cataract surgery. J refractive Surg (Thorofare NJ: 1995).

[27] Hashemi H, Khabazkhoob M, Soroush S, Shariati R, Miraftab M, Yekta A (2016). The location of incision in cataract surgery and its impact on induced astigmatism. Curr Opin Ophthalmol.

[28] Barequet IS, Yu E, Vitale S, Cassard S, Azar DT, Stark WJ (2004). Astigmatism outcomes of horizontal temporal versus nasal clear corneal incision cataract surgery. J Cataract Refract Surg.

[29] Eslami Y, Mirmohammadsadeghi A (2015). Comparison of surgically induced astigmatism between horizontal and X-pattern sutures in the scleral tunnel incisions for manual small incision cataract surgery. Indian J Ophthalmol.

[30] Kessel L, Andresen J, Tendal B, Erngaard D, Flesner P, Hjortdal J (2016). Toric intraocular lenses in the correction of Astigmatism during cataract surgery: a systematic review and Meta-analysis. Ophthalmology.

[31] Chang JSM (2018). Femtosecond laser-assisted astigmatic keratotomy: a review. Eye and vision (London England).

[32] Nagpal R, Sharma N, Vasavada V, Maharana PK, Titiyal JS, Sinha R (2015). Toric intraocular lens versus monofocal intraocular lens implantation and photorefractive keratectomy: a randomized controlled trial. Am J Ophthalmol.

[33] Abu-Ain MS, Al-Latayfeh MM, Khan MI (2022). Do limbal relaxing incisions during cataract surgery still have a role?. BMC Ophthalmol.

[34] Ali MA, Kobashi H, Kamiya K, Igarashi A, Miyake T, Elewa ME et al. Comparison of astigmatic correction after femtosecond lenticule extraction and wavefront-guided LASIK for myopic astigmatism. Journal of refractive surgery (Thorofare, NJ: 1995). 2014;30(12):806 – 11. 10.3928/1081597X-20141113-03 25437478

[35] Rubenstein JB, Raciti M (2013). Approaches to corneal astigmatism in cataract surgery. Curr Opin Ophthalmol.

[36] Hirnschall N, Gangwani V, Crnej A, Koshy J, Maurino V, Findl O (2014). Correction of moderate corneal astigmatism during cataract surgery: toric intraocular lens versus peripheral corneal relaxing incisions. J Cataract Refract Surg.

[37] Felipe A, Artigas JM, Díez-Ajenjo A, García-Domene C, Alcocer P (2011). Residual astigmatism produced by toric intraocular lens rotation. J Cataract Refract Surg.

[38] Potvin R, Kramer BA, Hardten DR, Berdahl JP. Toric intraocular lens orientation and residual refractive astigmatism: an analysis. Clinical ophthalmology (Auckland, NZ). 2016;10:1829–36. 10.2147/OPTH.S114118 PMC5036610 27703323

